# Outbreak of scabies among preschool children, Accra, Ghana, 2017

**DOI:** 10.1186/s12889-019-7085-6

**Published:** 2019-06-13

**Authors:** Basil Benduri Kaburi, Donne Kofi Ameme, George Adu-Asumah, Dora Dadzie, Emmanuel Kwame Tender, Smith Vincent Addeh, Theophilus Aryee, Adolphina Addo-Lartey, Samuel Oko Sackey, Fredrick Wurapa, Edwin Andrew Afari, Ernest Kenu

**Affiliations:** 1 Ghana Field Epidemiology and Laboratory Training Programme, School of Public Health, University of Ghana, Legon, Ghana; 2 Department of Epidemiology and Disease Control, School of Public Health, University of Ghana, Legon, Ghana

**Keywords:** Scabies, Outbreak investigation, Benzyl benzoate, Preschool children, Ghana

## Abstract

**Background:**

Scabies occurs worldwide with a prevalence between 0.3 and 46.0%. In Ghana, even though a 5.1% proportion of scabies was reported in a retrospective review of skin diseases at the Korle Bu Teaching Hospital, the nationwide prevalence of scabies is unknown. Overall, its burden is higher in tropical regions. Scabies outbreaks mostly occur among children, the elderly in nursing homes, and prison inmates. Even though primary scabies hardly results in mortalities, the pain, itch, and systemic complications from secondary bacterial infections account for about 1.5 million years lived with disabilities. We investigated a scabies outbreak among school children in Ghana to determine its magnitude, stop the outbreak, and institute preventive measures to minimize risks of future outbreaks.

**Methods:**

The investigation was conducted between March 14 and May 17, 2017 among pupils of Presbyterian Secondary Staff Basic School in Accra. We defined a case as a school child who on clinical examination, had an intensely pruritic rash on at least one typical predilection site with or without a burrow, or positive skin scrapings on microscopy. We screened and line listed cases, performed laboratory investigations on skin scrapings and wound swaps, and conducted an environmental assessment. We performed descriptive statistics on data, and calculated attack rate ratios (ARR) at 95% confidence level.

**Results:**

Of 823 preschool children screened, 92 were cases. Median age of cases was 4 years (range 2–7 years) and their modal age was 3 years. The overall attack rate was 11.2% (92/823). The sex specific attack rate was 11.5% for males, and 10.8% for females (ARR: 0.93; CI: 0.67–1.28). Compared with the least affected class (crèche), the nursery one class was worst affected (ARR: 5.14; CI: 3.44–7.50). On microscopy, all skin scrapings were negative for scabies. *Staphylococcus aureus* and *Streptococcus spp.* were isolated from secondarily infected scabies lesions.

**Conclusions:**

A scabies outbreak with a propagated source occurred among preschool children. The 3-year-old pupils were most affected. It was controlled by mass treatment with benzyl benzoate and health education. Classrooms and sleeping mats were disinfected. We recommended the decongestion of classrooms and discouraged sharing of sleeping mats.

**Electronic supplementary material:**

The online version of this article (10.1186/s12889-019-7085-6) contains supplementary material, which is available to authorized users.

## Background

Scabies is an ectoparasitic infestation caused by the mite *Sarcoptes scabiei* var. *hominis.* It is considered one of the neglected tropical diseases, and presents as various skin lesions that are intensely pruritic, and spread by person-to-person contact [1]. According to the World Health Organisation (WHO), scabies affects people from every country [1]. Scabies is ubiquitous, but presents a major public health threat to developing countries in particular [1]. Young children and the elderly in resource-poor communities are especially susceptible to scabies [2]. The highest rates occur in hot tropical climates, where infestation is endemic with sporadic outbreaks [3]. Poverty and overcrowding also promote the spread of scabies [4]. Globally, the prevalence of scabies varies from 0.3 to 46%; with an estimated 1.5 million years lived with disability [1]. In Ethiopia, the weighted prevalence of clinically confirmed skin diseases is 22.5% with scabies as the most common diagnosis [5]. The prevalence of scabies among school children in Nigeria is about 4.7% [6]. In Ghana, even though a 5.1% proportion of scabies was reported in a retrospective review of skin diseases at the Korle Bu Teaching Hospital, the nationwide prevalence of scabies is unknown [7].

Owing to its acute and secondary complications, scabies poses a significant but mostly under-recognized socioeconomic burden to affected persons and communities [8, 9]. Its characteristic intense pruritus severely affects sleep, work, and the quality of life [10]. Excoriations resulting from scratching expose the skin to secondary bacterial infections that usually result in pyoderma, impetigo, and cellulitis. Among scabies patients, an annual global estimate of 660,000 incident cases of secondary bacterial infections are caused by *Streptococcus pyogenes* alone; with a case fatality rate of about 24.2% [11]. Long term sequelae of these acute secondary bacterial infections include acute post-streptococcal glomerulonephritis (APSGN) and rheumatic heart disease. Skin infections are responsible for approximately half of APSGN cases in tropical settings - estimated at more than 470,000 cases per year [12]. Scabies and its complications impose a significant socioeconomic burden on affected persons, immediate families and communities, as well as healthcare systems. Direct financial costs of scabies relate to the cost of medicines, loss of jobs and job opportunities, and institutional outbreaks resulting from hospitalization of cases [13].

These disease-burden ramifications of scabies infestation notwithstanding, it is not considered a priority disease in Ghana. Indeed, the most recent five-year strategic plan (2013–2017) for reducing the burden of neglected tropical diseases in Ghana does not include scabies [14]. This is not surprising because WHO had just adopted scabies as a neglected tropical disease at the tenth meeting of its Strategic and Technical Advisory Group for Neglected Tropical Diseases held on 29th and 30th March 2017 [15]. This decision was taken at the time of this outbreak investigation and it is yet to reflect in the disease control strategic plan of Ghana. Data on scabies in general, and published information in particular, are scarce. Even though scabies is considered an endemic skin disease in many rural and urban poor communities of Ghana, the evidence is largely anecdotal. Occasionally, sections of the Ghanaian media report outbreaks of scabies among inmates of some prisons in the country; the most recent of which was the Ho Central Prisons in 2016 [16].

On 14th March 2017, a community health nurse notified a resident of Ghana Field Epidemiology and Laboratory Training Programme (GFELTP) about her nursery one son who was taken ill of an intensely pruritic skin rash. The following day, the resident visited the school and found five other classmates of this child with similar lesions. The skin lesions were intensely pruritic erythematous papules on the face, groin, buttocks, and lower limbs that were laced with excoriations from repeated scratching. The class teacher indicated that because of similar skin lesions, two (2) other pupils had stopped attending school for some days before the resident’s visit. The GFELTP secretariat was notified about this suspected outbreak; and a team of field epidemiology residents was constituted to investigate the outbreak. We investigated the scabies outbreak to its magnitude, control and prevent future outbreaks.

## Methods

### Outbreak setting

The outbreak occurred in Presbyterian Secondary Staff Basic School in the La Nkwantanang Madina municipality of the Greater Accra Region. The total population of the school was 3674. The population of the preschool children was 845. The outbreak investigation was conducted from March 14 to May 17, 2017.

### Outbreak, and case definitions

The following definitions were used:

1. Outbreak definition: two or more consecutive cases of scabies among pupils/staff of Presbyterian Secondary Staff Basic School within 4–6-week period
2. Case definition: a pupil or staff of the Presbyterian Secondary Staff Basic School, with mites, mite eggs, or mite faeces identified in skin scraping; OR

A pupil or staff of the Presbyterian Secondary Staff Basic School with clinical signs and symptoms of scabies (rash, severe pruritus, burrows).

### Case finding and line listing

A line listing template was adopted from a scabies outbreak investigation report by the Department of Health, New Jersey, USA [17]. To find cases, physical examinations were conducted by a team of four GFELTP residents comprising: two physicians, one disease control officer, and a biologist. The team moved together from one class to the other and examined each pupil together – one performing the physical examination and the remaining observing closely. Based on the case definitions, at least, three of these four investigators had to agree on the clinical diagnosis of scabies for it to be added onto the line list. This approach was taken to improve inter and intra-operator reliability of examination findings.

Data of cases that were captured on the line list included: the name, age, class, place of residence, duration since onset, signs and symptoms (rash, burrows, itching, excoriation, crusting, presence of secondary bacterial infection) results of laboratory test, duration of treatment, and some risk factors (household size, number of baths per day, type of bedding, and hygiene practices).

### Laboratory investigations

We took two types of specimens: skin scrapings for the isolation of scabies mite, their eggs, or faeces; and wound swaps to identify agents responsible for secondary bacterial infection of scabies wounds.

First, we performed skin scrapings at the end of the burrows in non-excoriated and non-inflamed areas of scabies lesions unto filter papers using sterile scapel blades size# 15. We transferred scrapings from each site unto a microscopic slide, added 2 drops of 10% potassium hydroxide (KOH), mixed well, applied a cover slip, and ensured that no air bubbles were trapped within. We labeled these slides, placed them on cotton wool soaked in 10% KOH, transferred them into petri dishes, and then covered and sealed each with cello tape. We transported the petri dishes to the laboratory in a canister. Within seven hours of preparation, the biomedical scientist of our investigation team examined the slides under a light microscope for mites, their eggs, or faeces. Two other microbiologists cross examined the slides in turns. Negative samples were incubated for 72 for possible fungal growths.

Second, we took wound swaps from affected pupils with suppurating scabies sores. All wound swaps were inoculated into brain heart infusion (BHI) in 20% glycerol as transport medium. The samples were tripled-packaged and transported by reverse cold chain to the Ridge Hospital, Accra. All samples were plated on three media viz. chocolate agar (CA), blood agar (BA), and MacConkey agar. The inoculated CA and BA plates were incubated at 35 °C – 37 °C in 5–10% carbon dioxide for 24 h. The inoculated MacConkey agar plate was incubated at 37 °C for 24 h. All plates were observed for growth. We performed Gram staining on resulting colonies. For Gram positive cocci we performed catalase tests on the colonies to differentiate staphylococci from streptococci. For catalase positive isolates, we performed coagulase test to differentiate *Staphylococcus aureus* from coagulase negative staphylococci.

### Environmental assessment

We conducted an environmental assessment. This included an assessment of the state of classrooms, average class occupancy, seating arrangement, sleeping conditions during siesta, washroom hygiene, and the general state of cleanliness of the school premises.

### Data analysis

We conducted descriptive and inferential statistical analyses. Results of univariate analysis of categorical variables were expressed as frequencies and proportions; and results for continuous variables were expressed as median and range. We described the data in terms of time, place and person. We constructed an epidemic curve to show the onset, and illustrate the magnitude and mode of propagation of the outbreak.

We calculated the following: overall attack rate (AR), and specific attack rates for age and sex. Attack Rate Ratios (ARR) and their corresponding 95% confidence intervals (CI) were estimated to determine the association of scabies with possible predictors (e.g. sex, class, age). Data entry and cleaning was done using Microsoft excel version 2010. We used Microsoft excel version 2010 for the descriptive analysis, and STATA version 13.1 for the inferential analysis.

### Ethical considerations

This outbreak investigation was deemed a response to a public health emergency by the Ghana Health Service and hence did not require a formal review by Ethical Review Committees. Since all respondents were minors, a written consent was obtained from the Parents Teachers Association (PTA) of the school. All pupils who participated in the investigation accepted to be interviewed and examined without any form of coercion or inducement. The privacy of respondents during screening, and confidentiality of captured data were observed throughout the investigation. Data were kept under password and made available strictly on a need to know basis.

## Results

### Descriptive epidemiology

A total of 823 pupils were screened and all of them were preschool children (crèche to kindergarten 2). From these, 92 scabies cases were identified of whom 51.1% (47/92) were males. The median age of cases was 4 years (range 2–7 years), and the modal age was 3 years. The overall attack rate was 11.2% (92/823). There were no fatalities during the period of investigation.

**Fig. 1:**
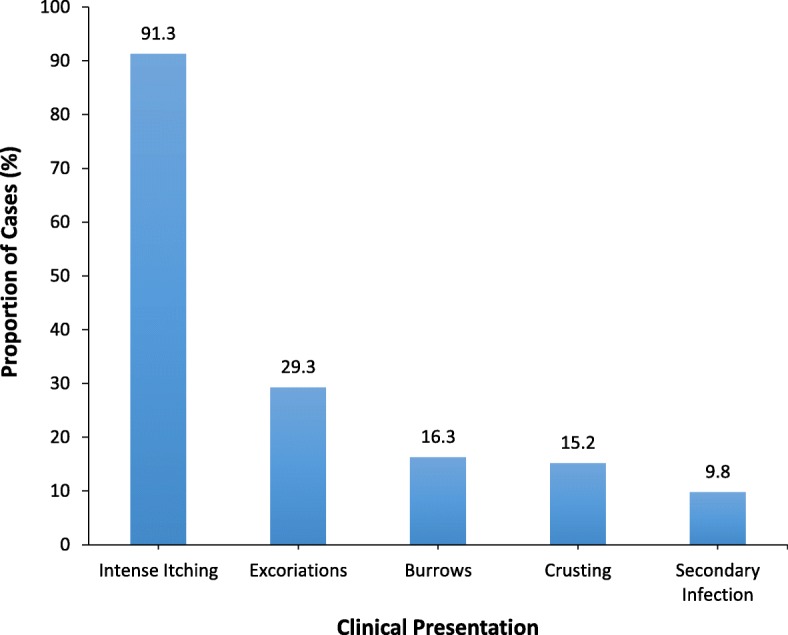
Clinical presentation of scabies among pupils, Presec Staff Basic School, 2017

**Fig. 2:**
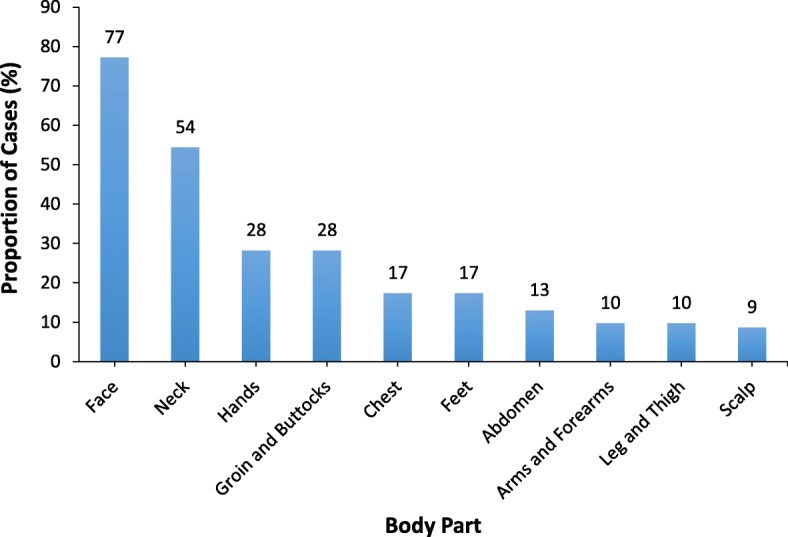
Distribution of scabies lesions by body part among pupils, Presec Staff School, 2017

The index case was a 3-year-old boy who was in the nursery one class. His mother is a community health nurse who noted that her son had developed pruritic rashes on the face and neck which did not respond to topical antibiotics for a period of one week. In the evening of March 14, 2017, she suspected scabies and placed a personal call to a GFELTP resident who is a medical officer to consult on her son’s condition. The next morning, the resident made a visit to the school, confirmed the diagnosis, and identified more cases in the pupil’s class. Affected pupils presented with typical pruritic lesions of scabies with excoriations from repeated scratching. Cases presented with typical signs and symptoms of scabies (Fig. 1). The worst affected body part was the face (Fig. 2). Some of the lesions had developed suppurative secondary bacterial infections. Nearly 10% of cases had pyoderma from secondary bacterial infections.

**Fig. 3:**
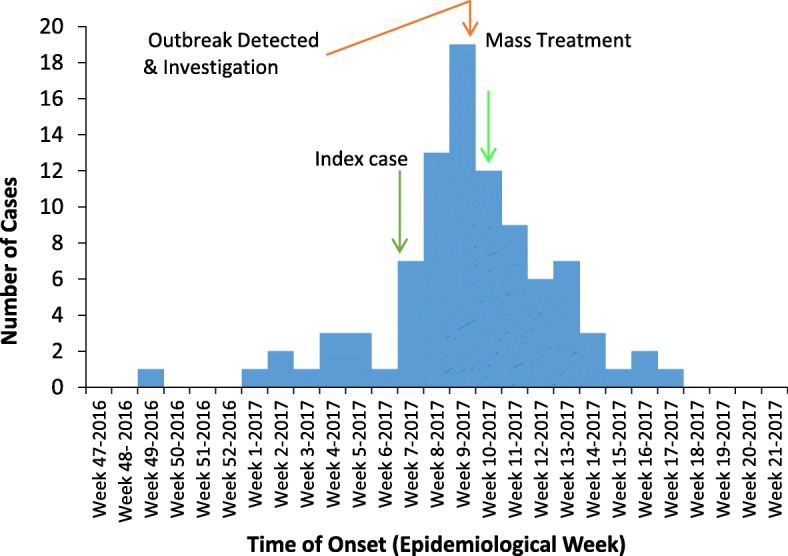
Epidemic curve of scabies outbreak, Presec Staff School, 2017

The epidemic curve showed a propagated source for the outbreak (Fig. 3). The earliest case captured in the investigation (a likely primary case) occurred in the epidemiological week 49 of 2016. The index case developed the scabies in the epidemiological week 7 of 2017. The number of cases rose gradually from epidemiological week 1 of 2017 until epidemiological week 7 when the numbers rose sharply; peaking in epidemiological week 9. The last case occurred in epidemiological week 18 of 2017.

### Analytic epidemiology

The sex specific attack rate was 11.5% for males, and 10.8% for females (Table 1). However, the risk of attack did not differ by sex (ARR: 0.93; CI: 0.67–1.28). Compared with the 2-year-old pupils, the 3-year-old pupils were more likely to develop scabies (ARR: 4.77; CI: 3.18–6.98). Using the least affected class (crèche) as the reference class, the worst affected class was the nursery 1(ARR: 5.14; CI: 3.44–7.50).

**Table 1:** Demographic characteristics of pupils and their association with the scabies, Presbyterian Secondary Staff School, 2017

Variable	Cases	Non-Cases	Total	Attack Rate (%)	Attack Rate Ratio	95% CI
Sex						
Male	47	361	408	11.52	1	
Female	45	370	415	10.84	0.93	0.67–1.28
Age (Years)						
2	4	92	96	4.17	1	
3	33	159	192	17.19	4.77	3.18–6.98^a^
4	18	195	213	8.45	2.12	1.23–3.44^a^
5	24	170	194	12.37	3.24	2.02–5.00^a^
6	11	88	99	11.11	2.88	1.38–5.40^a^
7	2	27	29	6.90	1.7	0.19–6.78
Class						
Crèche	4	92	96	4.17	1	
Nursery 1	34	152	186	18.28	5.14	3.44–7.50^a^
Nursery 2	18	164	182	9.89	2.52	1.46–4.12^a^
Kindergarten1	22	156	178	12.36	3.24	1.98–5.09^a^
Kindergarten 2	14	167	181	7.73	1.92	1.03–3.33^a^

^a^Statistically significant associations

### Laboratory findings

Eight skin scraping were taken from cases, and all tested negative by microscopy at the Ridge Hospital microbiology laboratory, Accra. The incubated negative skin scrapings yielded no fungal growths. Of 11 wound swaps collected and incubated, 9 of them showed mixed growth and 2 showed pure growth. On Gram stain, the two pure growths were Gram positive cocci in chains; and these were identified as streptococcus species. Gram reactions on the plates with mixed growth showed Gram positive diplococcic and Gram positive cocci in clusters. From these plates, some isolates tested negative for catalase and were identified as streptococcus species. From these same plates, some isolates tested positive for catalase, and were identified as staphylococcus species. Among these isolates of staphylococcus species, some isolates tested positive for coagulase and were identified as *Staphylococcus aureus*.

### Environmental findings

The school compound was walled. The immediate grounds to the classrooms were paved with concrete blocks. In the crèche and nursery classes, pupils shared sleeping mats during siesta. Each class from the nursery level and higher, was subdivided into four; each with an average of about 45 pupils. Each class had running water from a Veronica bucket for routine hand washing. The school compound and classrooms were clean. The school had washrooms with running water. However, water supply was not constant, and the facilities were under pressure from the large population of pupils.

## Discussion

We investigated a scabies outbreak affecting preschool children aged 2–7 years old in the Greater Accra Region of Ghana. Of these six ages, the 3-year-old children were worst affected (36%). Unlike the economically advanced countries where rates of scabies infestation are similar across age groups [18], in developing countries, persons in their preschool to adolescent ages are disproportionately affected [19, 20]. The occurrence of this outbreak among preschool children in our study underscores the increased risk of scabies in this age group. The disproportionate prevalence of scabies among young children, possibly reflects both increased exposure and, in endemic situations, lack of immunity [4]. Extant epidemiological evidence has it that, scabies affects both sexes similarly and our findings support these reports [21]. Scabies outbreaks often occur in institutions such as hospitals, nursing homes, prisons, or kindergartens [4]. The occurrence of this outbreak among preschool children from crèche to kindergarten underscores the increased risk of scabies outbreaks among this sub-population.

Some socio-demographic patterns in scabies epidemiology are related to differences in overcrowding, housing, socioeconomic, and behavioural factors [4]. The average class size was 45 pupils. Due to the large numbers, pupils sat in pairs. This may have facilitated person to person contact and could partly explain the propagated mode of spread of this outbreak. Pupils also shared sleeping mats during siesta. In an epidemiologic and therapeutic reassessment of scabies by Burkhart and colleagues, the sharing of intimate objects such as water closets, and fomites such as sleeping mats and beddings are major risk factors in the transmission of scabies [19]. This was likely a contributory factor to the propagation of the outbreak. There is limited evidence that access to hand hygiene or water significantly contributes to the spread of scabies. However, in their study of skin infections and infestations in Aboriginal communities in northern Australia, Currie and Carapetis reported that, the acquisition and spread of scabies is also associated with poor hygiene [22]. In our study, the poor sanitary conditions of the overburdened washrooms might just partly explain the spread of this outbreak. Overall, the literature is inconclusive on the role of poor hygiene as a significant risk factor for the acquisition and spread of scabies. Instead, poor hygiene has been found to be significantly associated with secondary bacterial infections; and that, improved hygiene using water and soap has been shown to reduce the prevalence of impetigo among scabies patients [23]. In our investigation therefore, the overcrowding of classrooms and sharing of sleeping mats appear as more tangible contributory factors to the spread of the outbreak.

### Secondary bacterial infections and complications

In our study, nearly 10% of the cases presented with pyoderma. The intense pruritus typical of scabies is caused by a hypersensitive reaction to components of the saliva, eggs, and faecal material of the scabies mites. Repeated scratching results in excoriation of the epidermis. These excoriations serve as entry points for both skin commensals and pathogenic bacteria. Pyoderma is therefore a common complication especially among scabies patients in the tropics who have multiple lesions [24]. In 1997, a study of the prevalence of secondary infections among scabies patients in Ghana revealed that, of 110 patients who developed secondary infections, culture results showed a mixture of both aerobic and anaerobic bacteria. *Staphylococcus aureus* was the most isolated aerobic bacteria species (39.1%), and *Pepostreptococcus spp.* was the most isolated anaerobic bacteria species (14.2%) [25]. Similarly, in our study, *Staphylococcus aureus* and *streptococcus spp.* were identified as the main agents causing secondary bacterial infection among pupils with suppurative scabies sores. If streptococcal skin infections are not effectively treated, APSGN can result. In Trinidad, the number of nephritogenic strains of streptococci isolated increased with a rise in the occurrence of scabies and APSGN in the general population [12, 25]. Lawrence and colleagues showed that, the control of scabies and the associated streptococcal infection of scabies sores among children significantly reduces haematuria [26]. We infer that, the significant reduction of haematuria in their study resulted from the reduction in renal damage usually caused by APSGN – a complication of streptococcal infection of the scabies sores. Outbreaks of APSGN coincide with those of scabies, and asymptomatic renal disease is also common [27, 28]. These insults to the kidney in childhood; as in the case of these 92 preschool children, contribute to the development of chronic kidney disease and subsequent renal failure in adulthood [27, 29].

### Diagnosis: clinical and laboratory methods

The laboratory diagnosis of scabies requires the microscopic detection of the mite, ova, or faecal pellets. However, in our study, all the skin scrapings examined with light microscopy turned out negative for mite, their eggs, or faeces. This method is very specific, and a mite or eggs seen under the microscope are diagnostic. We diagnosed cases using typical clinical signs and symptoms. In resource limited settings like Ghana, clinical diagnosis is the more practical way of diagnosing scabies because the sensitivity of skin scrapings is so low that its diagnostic usefulness is questionable [4]. In their study of the comparison of dermoscopy, skin scraping, and the adhesive tape test for the diagnosis of scabies in a resource-poor setting in northeast Brazil, Walter and his colleagues found that the sensitivity of skin scraping was low (0.46; 95% CI, 0.31–0.62) and concluded this method cannot be recommended as a diagnostic tool in such setting [30]. In the light of these challenges, a practical approach for the diagnosis of scabies that includes the presence of papules, vesicles, pustules, itching (especially at night), and a positive family history as proposed by Heukelbach and colleagues is an acceptable method for the diagnosis of a case [21, 31]. These evidence based practicalities informed the clinical approach we used for case identification in our investigation of the outbreak. Epiluminescence microscopy and high-resolution videodermatoscopy seem to be promising diagnostic methods [32, 33]. Even then, there have been no robust studies to compare the sensitivity and specificity of clinical diagnosis and microscopic examinations of the skin. The high equipment cost means that, the techniques are accessible only in well-resourced settings. Thus, the diagnosis of scabies and hence, the detection of outbreaks would remain largely clinical in Ghana for the time being.

### Study limitations

Given the ages of the affected pupils, they were unable provide detailed clinical history of their condition. Many parents were hard to reach; both at home (via home-visits) and by telephone calls, to assist with details of the disease progression. We were also unable to carry out contact tracing in the communities within which affected pupils resided.

### Public health actions taken

We carried out mass treatment of pupils for scabies using benzyl benzoate cream. We referred pupils with scabies sores to the municipal hospital for further management. We conducted health education on scabies for staff and pupils of the school. As part of the continual health education, we posted pictures showing the clinical presentations of scabies on all school notice boards to raise awareness and help improve surveillance of scabies within the school. We supervised the cleaning and disinfection of classrooms, sleeping mats, and washrooms. School authorities welcomed our recommendation to stop the practice where pupils share sleeping mats at school.

## Conclusions

A scabies outbreak affecting preschool children occurred in the Presbyterian Secondary Staff Basic School in Accra. The outbreak was determined to have a propagated source. Scabies lesions affected all body parts and the distribution was typical – worse in the face, hands, the groin and buttocks. Several factors including overcrowding of classrooms, sitting in pairs, and sharing sleeping mats contributed to the spread of the outbreak. Multiple measures including mass treatment with benzyl benzoate cream, health education, and disinfection of sleeping mats contributed to control of the outbreak.

## Additional file

Additional file 1: Dataset for scabies outbreak. (XLSX 53 kb)

## Data Availability

All data generated or analysed during this study are included in this published article [and its Additional file 1].

## Abbreviations

APSGN: Acute Post-streptococcal Glomerulonephritis
AR: Attack Rate
ARR: Attack Rate Ratio
BA: Blood Agar
BHI: Brain Heart Infusion
CA: Chocolate Agar
CI: Confidence Interval
GFELTP: Ghana Field Epidemiology and Laboratory Training Programme
KOH: Potassium Hydroxide
PTA: Parents Teachers Association
WHO: World Health Organization

## References

[1] World Health Organization | Scabies. Available at: https://www.who.int/neglected_diseases/diseases/scabies/en/. Accessed 21 Mar 2017.

[2] Badiaga S, Menard A, Dupont HT, Ravaux I, Chouquet D, Graveriau C, Raoult D, Brouqui P (2005). Prevalence of skin infections in sheltered homeless of Marseilles (France). European journal of dermatology..

[3] World Health Organization (2005). Epidemiology and management of common skin diseases in children in developing countries.

[4] Heukelbach J, Feldmeier H (2006). Scabies. The Lancet..

[5] Leekassa R, Bizuneh E, Alem A, Fekadu A, Shibre T (2005). Community diagnosis of common skin diseases in the Zay community of the Zeway Islands, Ethiopia. Ethiopian medical journal..

[6] Ogunbiyi AO, Owoaje EM, Ndahi A (2005). Prevalence of skin disorders in school children in Ibadan, Nigeria. Pediatric dermatology..

[7] Rosenbaum BE, Klein R, Hagan PG, Seadey MY, Quarcoo NL, Hoffmann R, et al. Dermatology in Ghana: a retrospective review of skin disease at the Korle Bu Teaching Hospital Dermatology Clinic. The Pan African medical journal. 2017:*26*. 10.11604/pamj.2017.26.125.10954 PMC5429409 28533848

[8] Engels D, Savioli L (2006). Reconsidering the underestimated burden caused by neglected tropical diseases. TRENDS in Parasitology..

[9] Feldmeier H, Heukelbach J (2009). Epidermal parasitic skin diseases: a neglected category of poverty-associated plagues. Bulletin of the World Health Organization..

[10] Jackson A, Heukelbach J, Júnior C, de Barros E, Feldmeier H (2007). Clinical features and associated morbidity of scabies in a rural community in Alagoas, Brazil. Tropical Medicine & International Health..

[11] Carapetis JR, Steer AC, Mulholland EK, Weber M (2005). The global burden of group A streptococcal diseases. The Lancet infectious diseases..

[12] World Health Organization. Dept. of Child, Adolescent Health. Handbook IMCI: integrated management of childhood illness: World Health Organization; 2005.

[13] Hay RJ, Steer AC, Engelman D, Walton S (2012). Scabies in the developing world—its prevalence, complications, and management. Clinical Microbiology and Infection..

[14] Ghana Neglected Tropical Diseases programme. Five-year Strategic Plan, 2013–2017. Available at: http://www.schoolsandhealth.org/Shared%20Documents/Ghana%20Neglected%20Tropical%20Disease%20Masterplan%202013-2017.pdf. [Accessed 17 Aug 2017]

[15] Report of the Tenth Meeting of the WHO Strategic and Technical Advisory Group for Neglected Tropical Diseases. 29–30 March 2017 WHO, Geneva. Available at: https://www.who.int/neglected_diseases/NTD_STAG_report_2017.pdf [Accessed 09 March 2019]

[16] Ghana News Agency (GNA). Ho Central Prisons is “scabies free”. Available at: www.ghananewsagency.org/health/ho-central-prisons-is-scabies-free%2D%2D104743 [Accessed 17 Aug 2017].

[17] New jersey department of health communicable disease service general guidelines for the control of outbreaks in school and child care settings Available at: https://www.nj.gov/health/cd/documents/topics/outbreaks/guidelines_for_outbreaks_in_school_sttings.pdf. Accessed 22 Mar 2017.

[18] Christophersen J (1978). The epidemiology of scabies in Denmark, 1900 to 1975. Arch Dermatol.

[19] Burkhart CG, Burkhart CN, Burkhart KM (2000). An epidemiologic and therapeutic reassessment of scabies. Cutis.

[20] dos Santos MM, Amaral S, Harmen SP, Joseph HM, Fernandes JL, Counahan ML (2010). The prevalence of common skin infections in four districts in Timor-Leste: a cross sectional survey. BMC infectious diseases.

[21] Heukelbach J, Wilcke T, Winter B, Feldmeier H (2005). Epidemiology and morbidity of scabies and pediculosis capitis in resource-poor communities in Brazil. British Journal of Dermatology.

[22] Currie BJ, Carapetis JR (2000). Skin infections and infestations in Aboriginal communities in northern Australia. Australasian journal of dermatology..

[23] Luby SP, Agboatwalla M, Feikin DR, Painter J, Billhimer W, Altaf A, Hoekstra RM (2005). Effect of handwashing on child health: a randomised controlled trial. The Lancet..

[24] Goldust M, Rezaee E, Hemayat S (2012). Treatment of scabies: Comparison of permethrin 5% versus ivermectin. The Journal of dermatology..

[25] Adjei O, Brenya RC (1997). Secondary bacterial infection in Ghanaian patients with scabies. East African medical journal..

[26] Lawrence G, Leafasia J, Sheridan J, Hills S, Wate J, Wate C, Montgomery J, Pandeya N, Purdie D (2005). Control of scabies, skin sores and haematuria in children in the Solomon Islands: another role for ivermectin. Bulletin of the World Health Organization..

[27] Walker SL, Lebas E, De Sario V, Deyasso Z, Doni SN, Marks M, Roberts CH, Lambert SM (2017). The prevalence and association with health-related quality of life of tungiasis and scabies in schoolchildren in southern Ethiopia. PLoS neglected tropical diseases..

[28] Poon-King T, Svartman M, Mohammed I, Potter E, Achong J, Cox R, Earle D (1973). Epidemic acute nephritis with reappearance of M-type 55 streptococci in Trinidad. The Lancet..

[29] Berrios X (1990). Epidemic outbreak of acute post streptococcal glomerulonephritis. Revista chilena de pediatria..

[30] Walter B, Heukelbach J, Fengler G, Worth C, Hengge U, Feldmeier H (2011). Comparison of dermoscopy, skin scraping, and the adhesive tape test for the diagnosis of scabies in a resource-poor setting. Archives of dermatology..

[31] Romani L, Koroivueta J, Steer AC, Kama M, Kaldor JM, Wand H, Hamid M, Whitfeld MJ (2015). Scabies and impetigo prevalence and risk factors in Fiji: a national survey. PLoS neglected tropical diseases..

[32] Micali G, Lacarrubba F, Lo GG (1999). Scraping versus videodermatoscopy for the diagnosis of scabies: a comparative study. Acta dermato-venereologica..

[33] Lacarrubba F, Musumeci ML, Caltabiano R, Impallomeni R, West DP, Micali G (2001). High-Magnification Videodermatoscopy: A New Noninvasive Diagnostic Tool for Scabies in Children. Pediatric dermatology..

